# 

**Published:** 1881-12

**Authors:** 


					﻿\V hole N UMBER,
CCCCXL1II
Dec., 1881.
Number 6,
Vol. M/.
THE
CHICAGO
MEDICAL
T ournal and Examiner
EDITORS:
N. S. DAVIS. M.D., LL.D. JAS. NEVINS HYDE, A.M., M.D.
DANIEL R. BROWER, A.M. M.D.
CHICAGO:
PUBLISHED BY THE CHICAGO MEDICAL PRESS ASSOCIATION,
188 Clark Street.
the Notice of this Journal amone Advertisements.
				

